# Exploring the Mechanism through which *Phyllanthus emblica* L. Extract Exerts Protective Effects against Acute Gouty Arthritis: A Network Pharmacology Study and Experimental Validation

**DOI:** 10.1155/2022/9748338

**Published:** 2022-04-11

**Authors:** Haolin Tao, Jingbin Zhong, Yingshi Mo, Wenbin Liu, Hui Wang

**Affiliations:** ^1^College of Traditional Chinese Medicine, Guangdong Pharmaceutical University, Guangzhou 510006, China; ^2^Guangdong Provincial Key Laboratory of Pharmaceutical Bioactive Substances, Guangdong Pharmaceutical University, Guangzhou 510006, China

## Abstract

Increased uric acid levels and inflammatory reactions are the main factors considered responsible for the development of gouty arthritis. *Phyllanthus emblica* L. (PEL) has several promising pharmacological properties, including anti-inflammation and antioxidation. However, only a few studies have investigated its use for treating acute gouty arthritis (AGA), and the mechanism of action of PEL has not yet been clarified. The aim of this study was to verify the protective effects of PEL against gout and explore its underlying mechanism through network pharmacology and animal experiments. The main active components of the extract from PEL including mucic acid, mucic acid lactone, gallic acid, ethyl hexyl phthalate, and glucose were identified by UPLC-ESI-qTOF-MS. Network pharmacological analysis results revealed 13 active compounds in PEL and 85 related targets for the treatment of gout. The core mechanism of action of PEL is mainly associated with inflammation-related pathways, including the HIF-1, PI3K-Akt, TNF, and NOD-like receptor signaling pathways. Previous studies revealed that the NOD-like receptor signaling pathway, especially the NLRP3 inflammasome, plays an important role in the pathogenesis of AGA; therefore, we mainly investigated the effect of PEL on the NLRP3/ASC/caspase-1 pathway in gout rats. In the animal experiments, PEL was shown to have a satisfactory antigout effect, as it effectively reduced uric acid (UA) and xanthine oxidase (XOD) levels. In terms of inhibiting AGA-associated inflammatory reactions, our results showed that PEL significantly decreased the expression of NLRP3 and caspase-1 in ankle synoviocytes as well as the levels of downstream inflammatory factors, such as TNF-*α*, IL-10, and IL-1*β* in serum. Moreover, the results of our study show that PEL reduced MMP13 expression in the ankle synovium. Overall, the results of this study indicate that PEL exerted a therapeutic effect against AGA. Reducing uric acid levels, inhibiting inflammation, and decreasing the expression of MMP13 may be responsible for the therapeutic effect of PEL, which suggests that PEL can be further developed as a drug for the treatment of gout.

## 1. Introduction

Gout is a metabolic disease caused by the deposition of monosodium urate (MSU) in joints or tissues, and hyperuricemia is the main risk factor for gout [1]. Abnormally elevated serum uric acid (UA) levels (≥408 *μ*mol/L or 6.8 mg/dL) caused by the excessive intake of purine or poor renal excretion of UA increases the risk of developing AGA. Epidemiological studies show that the global prevalence rate of gout is 0.1–10% [2]. In western developed countries, the prevalence rate of gout in recent years has been 3–6% in men and 1-2% in women [3], which is significantly higher than that in previous decades. Currently, the clinical treatments for acute gouty arthritis (AGA) mainly consist of reducing serum UA levels and alleviating inflammation. In addition, the key genes involved in uric acid excretion, such as *SLC2A9*, *ABCG2*, and matrix metalloproteinase 13 (*MMP13*), which are related to cartilage degradation, are expected to become the new therapeutic targets for AGA [4–6].

Although allopurinol is widely considered the first-line drug for the treatment of gout, it has notable side effects, such as rash, allergy, and kidney damage [7], and patients are still at risk of recurrence after treatment with allopurinol alone or in combination with other drugs. Tibetan medicine has a long history of application in the treatment of gouty arthritis. For example, “Triphala”, which is prepared by mixing fruits of *P. emblica*, *Terminalia chebula*, and *T. bellerica* in equal proportions, has achieved good clinical efficacy in the treatment of hyperuricemia and gout [8]. As one of the main ingredients of these prescriptions, *Phyllanthus emblica* L. (PEL) has been considered to own the potential to treat gout [9]. Modern pharmacological studies have shown that the phenolic acids of PEL, which mainly consist of gallic acid, have anti-inflammatory and UA-lowering properties [10]. Sumantran et al. found that PEL extract exerts protective and reparative effects on cartilage injury [11]. Sarvaiya et al. also revealed the antigout activity of PEL on potassium oxonate induced gout rat model [12] Besides, according to the research of Cen, the anti-inflammatory and analgesic effect of PEL is partly due to its capacity to inhibit the release of inflammatory cytokines [13]. However, to date, only limited studies have investigated the mechanism of action of PEL in the treatment of gout.

Chinese medicine is a complex system with multiple components, multiple targets, and synergistic interactions among components [14], and thus, it is extremely difficult to study its role. Network pharmacology provides a novel perspective and strategy for the research of traditional Chinese medicine. The purpose of this study was to investigate the therapeutic effect of PEL on AGA as well as explore its mechanism of action. To this end, we used network pharmacology to study the active substances of PEL, determine the targets of its active components and the relationship between effective components and diseases, and construct a component-target-pathway (CTP) diagram to examine the mechanism through which PEL exerts protective effects against AGA.

## 2. Materials and Methods

### 2.1. Animals

Specific-pathogen-free (SPF) male Sprague Dawley (SD) rats weighing 200 ± 20 g were provided by the Guangdong Medical Experimental Animal Center (Guangzhou, China) [experimental animal license: SYXK (Guangdong) 2017-0125]. All rats lived in the barrier system of the Experimental Animal Center of Guangdong Pharmaceutical University. The rats were fed with pure water and standard food and housed at 24 ± 2°C under a 12 h light/12 h dark cycle. All animal experiment procedures were approved by the Ethics Committee of the Animal Experiment Committee of Guangdong Pharmaceutical University (ethical license no. gdpulacspf2017380).

### 2.2. Plant Materials

PEL was obtained from Puning City, Guangdong Province, China, and identified by Associate Professor Hongyan Ma (College of Traditional Chinese Medicine, Guangdong Pharmaceutical University).

### 2.3. Preparation of Ethanol Extract of PEL

The ethanol extract of PEL was obtained as described previously [15]. Briefly, raw PEL was washed, enucleated, and dried, and 126.0 g of the raw material was weighed. Next, we added 1,890 mL 75% ethanol to a 2 L round bottom flask for ultrasonic extraction for 25 min, with a solid/material ratio of 1 : 15. The extraction temperature was 45.8°C, and the extract was filtered and extracted three times. The experiment was repeated twice, the filtrate was concentrated to 252 mL at 45°C using a rotary evaporator, and the ethanol extract of PEL with a concentration of 0.5 gmL^−1^ was obtained.

### 2.4. Model Preparation and Drug Therapy

One week after adaptive feeding, SD rats were weighed and divided into five groups (*n* = 9): Control, Model, Colchicine, low-dose PEL-treated (PEL-L), and high-dose PEL-treated (PEL-H). AGA models were established for the Model, Colchicine, PEL-L, and PEL-H groups, and the Control group was given the same amount of normal saline as the other groups.

The method used to establish the AGA model was as follows [16–18]: 3% potassium oxycyanate was prepared and injected intraperitoneally into the rats with a dosage of 1 mL·100 g^−1^ twice for 7 days. On day 4 after the beginning of modeling, the urate crystals were injected into the medial side of the tibiotarsal joint (ankle) of the rats, while they were under ether anesthesia. A small incision was created over the ankle on the dorsal side of the hind limb, and the needle was inserted into the medial side of the tendon of the tibialis anterior. Next, 0.05 mL of sodium urate solution (25 mg·mL^−1^) was injected into the articular cavity using a 21-gauge needle with its tip beveled to 45°, and the contralateral bulging of the articular capsule was used as the injection standard to establish the AGA model. After injection, the rats showed lameness and joint swelling and could not bear weight, which was an indicator of the successful establishment of the model. The Control group was injected with the same volume of normal saline at the same site.

The administration procedure was as follows: after the first injection of potassium oxycyanate, the drug was administered intragastrically for 7 days. The Control and Model groups were administered the same volume of normal saline, and the Colchicine group was administered 0.05 mg·100 g^−1^ colchicine. Referring to the Chinese Pharmacopoeia and related literature [19], the PEL-H group was fed PEL at a dose of 5 g·kg^−1^·d^−1^, while the low-dose group was fed PEL at a dose of 2.5 g·kg^−1^·d^−1^. Nine rats in each group were injected intraperitoneally with the crude drug dose corresponding to their daily body weight.

### 2.5. Component Analysis via High-Resolution UPLC-MS Analysis

#### 2.5.1. UHPLC-ESI-QTOF-MS Conditions

The ultraperformance liquid chromatography-tandem mass spectrometry was performed using an Ultimate 3000 UHPLC system with a WPS-3000 autosampler, coupled to a Q-Exactive Orbitrap MS spectrometer, which is combined with quadrupole ion selection and Orbitrap high-resolution scanning (Thermo Fisher Scientific, Waltham, MA, USA). The chromatographic separation was carried out on a Waters ACQUITY UPLC BEH C18 column (100 mm × 2.1 mm, 1.7 *μ*m particle size; Waters, Milford, CT, USA). Chromatographic separations were performed at 25°C employing a gradient elution using 0.1% formic acid in water (A) and 0.1% formic acid in acetonitrile (B) as mobile phase at a flow rate of 0.15 mL/min. The elution consisted of a gradient of 95% A, 0-1 min; 5%–95% A, 1–16 min; 95% A, 16–18 min; and 5% B, 18–20 min, and the initial condition was maintained for 5 min. The sample injection volume was 10 *μ*L.

For MS detecting, a Q-Exactive Orbitrap-MS spectrometer was fitted with a heated electrospray ionization (ESI) ion source in both negative and positive ionization mode at full-scan mode ranging *m*/*z* 100–1000. The optimal MS parameters were as follows: spray voltage −2.8 kV/+3.5 kV; sheath gas flow rate, 35 arbitrary units; auxiliary gas flow rate, 10 arbitrary units; capillary temperature, 320°C; and auxiliary gas heater temperature, 350°C. The resolutions of full scan and dd-ms2 were 70,000 and 35,000 FWHM (full width at half maximum), while their AGC targets were set as 3 × 106 and 1 × 105, with their maximum IT (the maximum injection time allowed to obtain the set AGC target) 100 and 50 ms, respectively. The stepped NCE (normalized collision energy) was set to 35 V for MS/MS acquisition.

#### 2.5.2. Structure Analysis Procedure

MassLynx V4.1 software (Waters Corporation) was used to process the data. Manual identification was performed to characterize the chemical constituents from PEL by comparing the exact mass and fragmentation pattern of the compounds that were previously reported in articles.

### 2.6. Pharmacology Research

#### 2.6.1. Degree of Ankle Swelling

Following the injection of MSU crystals, the width of the right ankle joint at different time intervals was measured using a vernier scale. The left and right diameters (*a*) and the anteroposterior diameter (*b*) of the ankle joint were measured before establishing the arthritis model, 12 h, 24 h, and 48 h after the administration of the MSU crystals [20]. The ankle joint volume was calculated using the following formula: ankle joint volume = 1/2  ×  ab^2^.

#### 2.6.2. Inflammation Index and Dysfunction Index

The progression of acute arthritis was evaluated by the macroscopic scoring of the ankle joint. Data were recorded prior to establishing the arthritis model and 24 h after the administration of the MSU crystals. The inflammation and dysfunction scores of the rats were visually determined by two independent observers.

The following criteria were used to score dysfunction [21]: 

  Grade 0 (0 points): there is normal gait and both feet are evenly grounded. 
  Grade 1 (1 points): toes are not unfolded and foot is slightly limp.
  Grade 2 (2 points): foot is bent and clearly limping and toes are on the ground.
  Grade 3 (3 points): foot is completely lifted off the ground and there is three-legged gait.

The following criteria were used to score inflammation [22]: 

  Grade 0 (0 points): ankle joint is normal without any inflammatory reaction. 
  Grade 1 (2 points): joints have erythema of skin, mild swelling, and visible bony marks.
  Grade 2 (4 points): joints are notably red and swollen, bony landmarks have disappeared, and swelling is limited to the joints. 
  Grade 3 (6 points): there is swelling outside the joint, the degree of inflammatory reaction is more severe, the ability of the foot to move is weakened, and the foot is often lifted off the ground.

#### 2.6.3. Blood and Tissue Sample Treatment

The blood and ankle synovium samples of rats were collected. Serum UA and XOD levels were measured using ultraviolet spectrophotometry; the levels of tumor necrosis factor (TNF)-*α*, interleukin (IL)-1*β*, and IL-10 in the serum were measured using ELISA. The expression of NLRP3, MMP13, and caspase-1 in the ankle joint synovium was detected by western blotting. The pathological changes in the ankle joint were observed by hematoxylin and eosin (HE) staining.

#### 2.6.4. HE Staining Analysis

The joint synovium tissue was incubated in 4% paraformaldehyde solution for 24 h and subjected to gradient alcohol dehydration and routine paraffin embedding. Next, the joint synovium tissue was sliced into 5 *μ*m sections, and pathomorphological changes were observed using a microscope after HE staining.

#### 2.6.5. Measurement of Serum UA and XOD Levels

The obtained blood samples were centrifuged for 15 min. The upper serum layer was obtained, placed in an EP tube, and stored at −80°C. Next, the serum UA and XOD levels of each group were measured using the UA Test Kit and XOD Assay Kit, respectively (Nanjing Jiancheng Bioengineering Institute, Nanjing, China), according to the manufacturer's instructions. The detection wavelength was 490 nm, and the absorbance of the sample (*A*), blank (*A*_*0*_), and standard (*A*_*1*_) holes was measured. The standard concentration was 400 *μ*mol/L (*C*). The serum UA levels were calculated using the following formula:

(1) $$\begin{array}{c} \text{UA concentration}\left(\mu \text{mol}\cdot {\text{L}}^{-1}\right)=\frac{\left(A-A_{0}\right)}{\left(A_{1}-A_{0}\right)}\times C. \end{array}$$

XOD level was calculated using the following formula:

(2) $$\begin{array}{c} \text{XOD level}\left(\frac{U}{L}\right)=\left(A-0.018\right)\times \frac{2.37}{\left(0.0126\times 2\right)}. \end{array}$$

### 2.7. Network Analysis

#### 2.7.1. Identification and Screening of Active Components of PEL

Information on all components of PEL was collected from the PubMed and Traditional Chinese Medicine Database and Analysis Platform (TCMSP) databases [23]. The molecular formula and CAS number of each compound were obtained from the PubChem database [24].

Among the analyzed components, the compounds which met the conditions of OB ≥30% and DL index ≥0.18 were selected as active substances to create the database of PEL compound information. Oral bioavailability (OB) is considered one of the most important pharmacokinetic parameters in the process of absorption, distribution, metabolism, and excretion [25]. OB values greater than or equal to 30% are usually considered high OB values; high OB values are important indicators for the determination the drug-like (DL) index of active compounds. As a qualitative concept used to evaluate molecular efficacy in drug design, the DL index is often used for the rapid screening of active compounds [26]. In the DrugBank database, the average DL index is 0.18, and compounds with DL indices greater than or equal to 0.18 are considered to have high DL properties.

#### 2.7.2. Prediction of Targets of Active Components of PEL

The similarity ensemble approach and BATMAN-TCM databases were used to predict the compounds screened (as described in Section 2.1), with “*Homo sapiens*” as the restriction condition [27]. Next, the potential targets of the components were obtained.

#### 2.7.3. Collection of AGA-Related Disease Targets

AGA-related targets were obtained from the GeneCards, DrugBank, and OMIM databases [28–30]. After obtaining the aforementioned AGA-related disease targets, we deleted the repeated targets and constructed the disease target information database. Next, the disease targets of AGA were matched with the component targets of PEL to obtain the overlapping targets.

#### 2.7.4. Construction of a Protein-Protein Interaction (PPI) Network

The Molecular Complex Detection (MCODE) plug-in was used to import the intersection targets, as described in Section 2.1, into the STRING database [31], and the targets with interaction values greater than 0.7 were selected to construct a PPI network. Next, the results were imported into the Cytoscape 3.7.0 software, and the degree value of the nodes in the network was analyzed by the Network Analyzer function to screen the core target. In the PPI network, the higher the degree value of the target, the more important its role.

#### 2.7.5. GO and KEGG Pathway Enrichment Analysis

“OFFICIAL-GENE-SYMBOL,” “*P* ≤ 0.05,” and “*Homo sapiens*” were chosen as limited conditions to obtain the data, and GO and KEGG pathway enrichment analyses were carried out using the DAVID database [32, 33]. The results were in descending order according to the enrichment degree of the target. Then, we screened the top 10 processes and pathways and visualized them.

#### 2.7.6. Construction of a CTP Network

PEL components, intersection targets, and important pathways were imported into Cytoscape 3.7.0 to construct the CTP network of PEL.

### 2.8. Mechanism Validation

#### 2.8.1. Measurement of TNF-*α*, IL-1*β*, and IL-10 Levels

TNF-*α*, IL-1*β*, and IL-10 levels in the serum of rats in each group were measured using the TNF-*α*, IL-1*β*, and IL-10 ELISA kits, respectively (Cusabio, Wuhan, China), according to the manufacturer's instructions.

#### 2.8.2. Western Blotting of the Ankle Joint Synovium

The tissue samples were lysed on ice for 30 min using 1 × RIPA lysis buffer (Thermo Fisher Scientific, Waltham, MA, USA) containing protease inhibitor (Roche, Basel, Switzerland). The proteins in each sample were separated using sodium dodecyl sulfate–polyacrylamide gel electrophoresis on 12% polyacrylamide gels and were blotted onto a polyvinylidene fluoride membrane (Millipore Corporation, Germany). The membrane was blocked with 5% BSA in PBS for 1 h. It was then immersed in the corresponding primary antibody at 4°C overnight. On the second day, the membrane was incubated with the relevant secondary antibody (1 : 2000; Beyotime, Shanghai, China). TBST was used to rinse the strips thrice (15 min/times) throughout the whole process. Then, the developer was added, and the protein strips were detected and analyzed using ImageJ software (NIH, Bethesda, MD, USA).

### 2.9. Statistical Analysis

Data analysis was performed using SPSS Statistics for Windows, version 16.0 (SPSS Inc., Chicago, IL, USA), or GraphPad Prism 6.0 software. Data are presented as the mean ± standard deviation (SD) from at least three independent experiments, and each independent experiment was repeated three times to obtain the mean. Normally distributed datasets were analyzed with the unpaired Student's *t*-test for two independent groups or paired *t*-test for two dependent groups, and the one-way analysis of variance (ANOVA) followed by the Bonferroni's multiple comparisons test was performed for more than three groups. For all statistical comparisons, *P* < 0.05 was considered statistically significant and denoted with one, two, and three asterisks when lower than 0.05, 0.01, and 0.001, respectively.

## 3. Results

### 3.1. Identification of the Compounds

**Figure 1:**
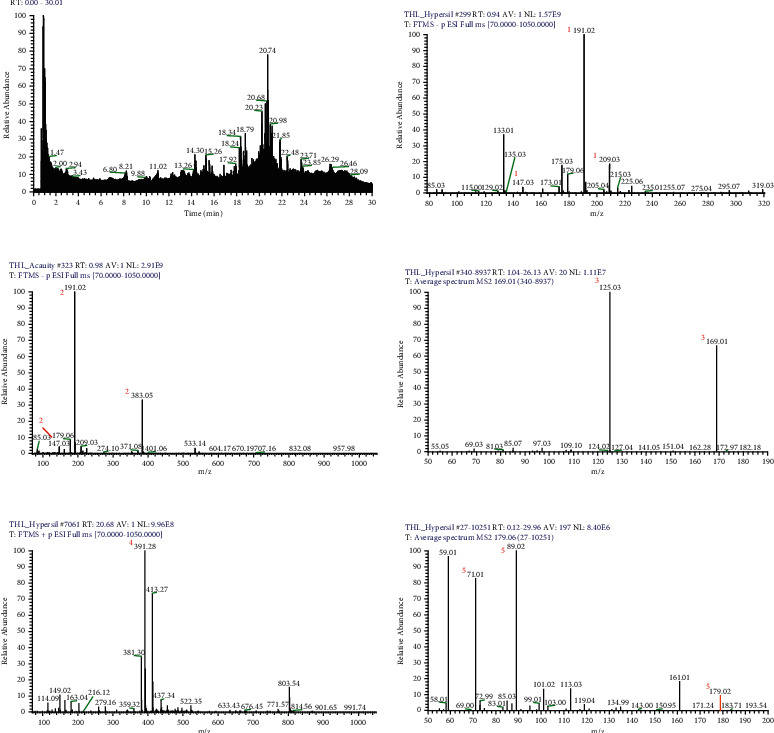
UPLC-MS^n^ chromatogram of the tannin fraction of PEL (a). Representative UPLC-QTOF-MS base peak chromatograms of mucic acid ((b) peak 1); mucic acid lactone ((c) peak 2); gallic acid ((d) peak 3); ethyl hexyl phthalate ((e) peak 4); and glucose ((f) peak 5).

UPLC-ESI-qTOF-MS chromatogram was employed to identify the main components in the ethanol extract of PEL. The total ion chromatogram profile of the tannin fraction of PEL was presented in both negative and positive ion modes, as shown in Figure 1(a). The possible structures of 5 peaks were deduced as shown in Figures 1(b)–1(f)). Under the optimized MS conditions, the negative and positive ion modes were used to identify the peaks of 5 main compounds including gallotannins, glucose, and phthalates. Data for all of these compounds are summarized in Table 1.

### 3.2. Pharmacology Research

#### 3.2.1. Protective Effect of PEL against Gout in Rats

**Figure 2:**
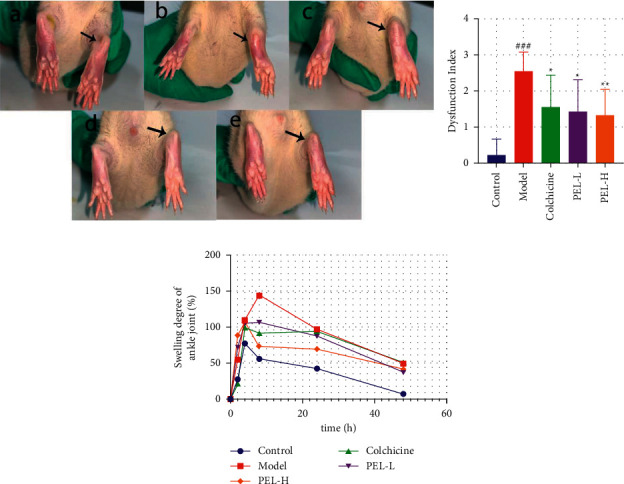
Changes in ankle joints of rats in each group after treatment (x̄ ± *s*, *n* = 9). (a) Ankle morphology of rats in each group, including a for Control group, b for Model group, c for Colchicine group, d for PEL-L group, and e for PEL-H group. The arrow indicates the injection site of MSU. (b) Step score of rats in each group. (c) Joint swelling degree of rats in each group. Compared with the blank group, ^###^*P* < 0.001; compared with the model group, ^*∗*^*P* < 0.01, ^*∗∗*^*P* < 0.05.

We first confirmed the therapeutic effect of PEL on gout. The appearance of ankle joint was observed 24 h after MSU injection (Figure 2(a)). Compared with the Model group, the ankle joint swelling in the Colchicine group improved significantly, and various symptoms, such as redness, swelling, and fever, disappeared. Compared with the Model group, the PEL-L and PEL-H groups experienced significant improvements in terms of ankle swelling and redness and fever elimination, but there was no significant difference between the two different concentration groups.

As shown in Figure 2(b), the dysfunction index of gout rats was significantly lower in the PEL-H group than in the Model group (*P* < 0.05). The ankle joint diameters of the rats in each group were measured at 0 h, 2 h, 4 h, 8 h, 24 h, and 48 h after MSU injection, and the degree of ankle swelling was calculated at each time point (Figure 1(c)). The ankle joint swelling degree in the Model group continued to increase 4 h after MSU injection; however, the ankle joint swelling degree of the Control, Colchicine, PEL-L, and PEL-H groups began to decrease. The ankle joint swelling degree was lower in both PEL groups than in the Model group at 8 h, 12 h, 24 h, and 48 h after injection. Collectively, our results revealed that PEL could reduce joint swelling in acute gout rats, and its effects began to appear 4 h after MSU injection.

#### 3.2.2. PEL Reduced the Levels of Serum Uric Acid in Gout Rats

**Figure 3:**
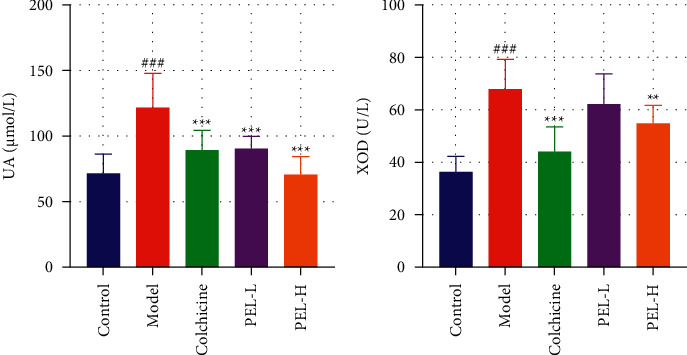
Changes in UA and XOD in serum of rats in each group after treatment (x̄ ± *s*, *n* = 9). (a) Rat serum UA value. (b) Rat serum XOD activity value. Compared with the blank group, ^###^*P* < 0.001; compared with the model group, ^*∗∗*^*P* < 0.01, ^*∗∗∗*^*P* < 0.001.

As the high uric acid levels are often an important factor accounting for gout, we studied the effect of PEL on uric acid next. The results showed that PEL significantly decreased serum UA levels in gout rats (Figure 3(a), *P* < 0.001), indicating that PEL exerts a positive effect on reducing uric acid levels. In addition, XOD activity decreased significantly in the PEL-H group; this activity also decreased in the PEL-L group but not significantly (Figure 3(b), *P* < 0.001).

#### 3.2.3. PEL Attenuates Ankle Joint Inflammation in Gout Rats

**Figure 4:**
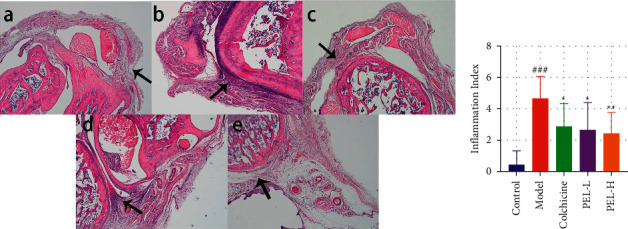
Changes in pathological tissue and inflammation of ankle joints of rats in each group after treatment. (a) Paraffin pathological sections of rat ankle joints stained by HE (×200), in which a is Control group, b is Model group, c is Colchicine group, d is PEL-L group, and e is PEL-H group. The arrow refers to the site of severe inflammation or therapeutic effect. (b) Inflammation index score of rats 24 h after MSU injection. Compared with the blank group, ^###^*P* < 0.001; compared with the model group, ^*∗*^*P* < 0.05, ^*∗∗*^*P* < 0.01.

Then, we investigate the protective effect of PEL against AGA which is mainly caused by the deposition of MSU. As shown in Figure 4(a), in the Control group, the structure of the ankle and its surrounding tissue was normal and clear, without any histopathological changes. The synovial epithelium was intact, and there was no inflammatory cell infiltration. In the Model group, inflammatory cells infiltrated the synovium and surrounding tissues. Pathological changes, such as synovium hyperemia, joint structure disorder, or incomplete joint structure, can be observed in the Model group. In the Colchicine group, the infiltration of inflammatory cells improved significantly, but pathological changes were still observed, such as a disordered and incomplete arrangement of joint structures. A small amount of inflammatory cell infiltration was observed in the PEL-L group, which was improved compared with the model group. In the PEL-H group, the joint structure was intact, and there was no exudate, dilatation, and hyperemia of the surrounding soft tissue vessels in the joint cavity. Compared with the model group, different doses of PEL could reduce synovial hyperplasia and inflammatory cell infiltration to different degrees. As shown in Figure 3(b), PEL significantly reduced the inflammatory response of ankle joints in gout rats.

### 3.3. Network Pharmacological Analysis

#### 3.3.1. Active Compounds in PEL

Network pharmacological analysis was used to further investigate the mechanism of anti-inflammatory effect. A total of 93 PEL compounds were obtained through literature search and the TCMSP database. After pharmacokinetic determination, 13 active compounds were obtained by setting OB ≥30% and DL ≥0.18. The component noted with “^*∗*^” was included in the construction of PEL active compound database owing to its strong biological activity and clear pharmacological effect. The results are shown in Table 2.

#### 3.3.2. Acquisition of Disease Targets for AGA and Intersection Targets

**Figure 5:**
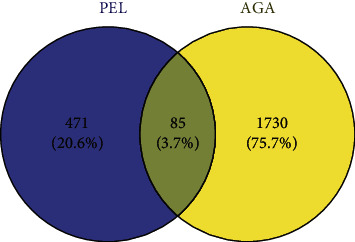
Venny diagram of ingredients-disease intersection targets. There are 471 targets of ingredients (left), 1730 disease targets (right), and 85 ingredients-disease intersection targets (middle).

In total, 556 gene targets of PEL active components were obtained from the similarity ensemble approach and BATMAN-TCM databases. We screened 1,815 possible AGA-related targets by using the keywords “Gout” and “Acute gouty arthritis” in the GeneCards, DrugBank, and OMIM databases. In total, 85 intersection targets were obtained by pairing the AGA-related targets with the component targets of PEL, and the Wayne diagram of the intersection targets was constructed using Venny 2.1.0 (Figure 5).

#### 3.3.3. Construction of PPI Network

**Figure 6:**
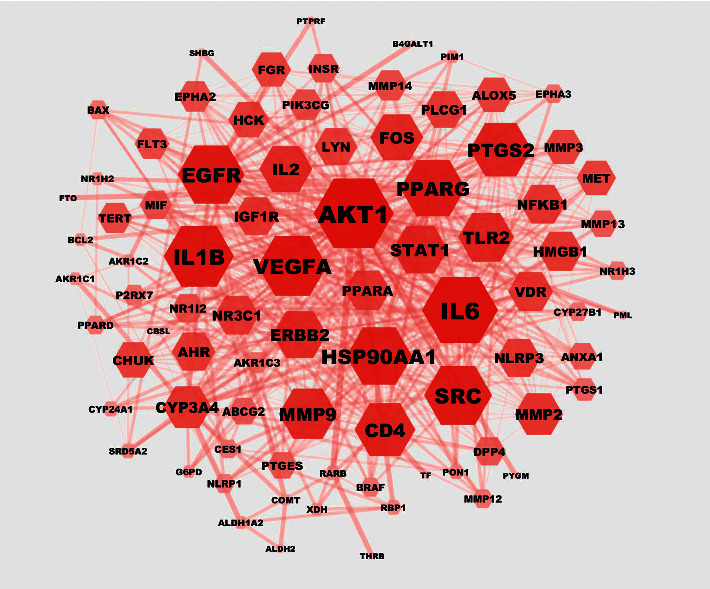
Network of protein-protein interactions. The hexagon represents the gene target, and the color depth and area size are positively correlated with the node value. Darker color and bigger size reflected the higher degree value.

The intersection targets were analyzed using the STRING database, and the PPI network diagram was constructed using Cytoscape 3.7.0 to analyze the results (Figure 6). The nodes represented the screened active components and targets, and the connections between the nodes represented the interactions between these bioanalyses. The node value represented the number of connections between molecules and targets in the core architecture of the network. In the PPI network, the larger the degree value of the node is, the more critical the biological role it may play [34]. The PPI network showed 170 nodes and 1,105 edges, with an average node value of 13. Among them, *Akt1* (degree value: 55), *Il6r* (degree value: 51), and *Il1b* (degree value: 45) were considered the hub genes in the PPI network.

#### 3.3.4. GO and KEGG Enrichment Analyses

**Figure 7:**
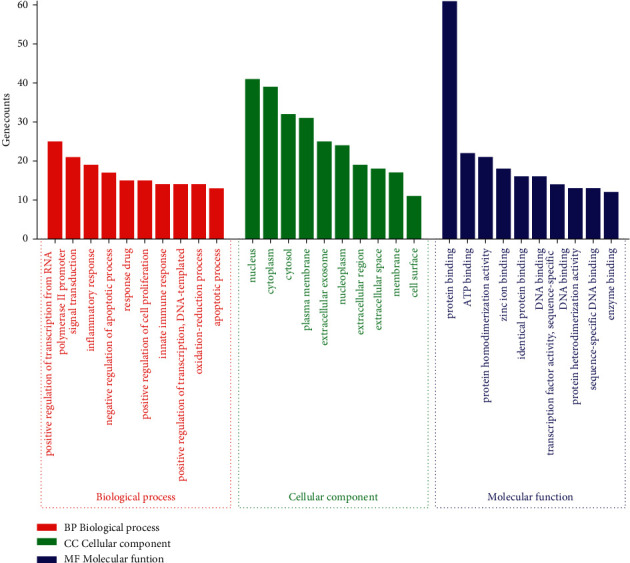
GO enrichment analysis of intersection targets.

Gene ontology (GO) enrichment analysis was used to further investigate the intersection targets, and the pathways involved in the top 10 intersection targets were constructed in a GO enrichment analysis path map (Figure 7): (1) 347 biological processes (BP) were involved in obtaining the active components of PEL, mainly comprising the positive regulation of transcription from RNA polymerase II promoter, signal transduction, and inflammatory response; (2) 47 related cellular components (CC) were obtained, including the nucleus, cytoplasm, and cytosol; and (3) 78 molecular functions (MF) related to the intersection targets were enriched, including protein binding, ATP binding, and protein homodimerization activity.

**Figure 8:**
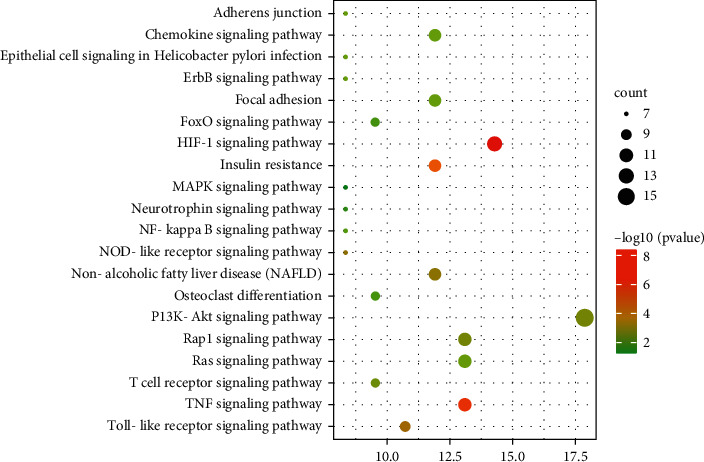
KEGG path enrichment diagram of intersection targets. The *X* axis shows the count of enriched genes, *Y* axis shows the KEGG pathways, and color represents the adjusted *P* value.

Kyoto Encyclopedia of Genes and Genome (KEGG) enrichment analysis was used to determine the signaling pathways related to anti-AGA, and 20 pathways were selected to map according to the number of genes involved. The main pathways were the PI3K-Akt, HIF-1, TNF, and NOD-like receptor signaling pathways. The results are shown in Figure 8.

#### 3.3.5. Construction of a CTP Network

**Figure 9:**
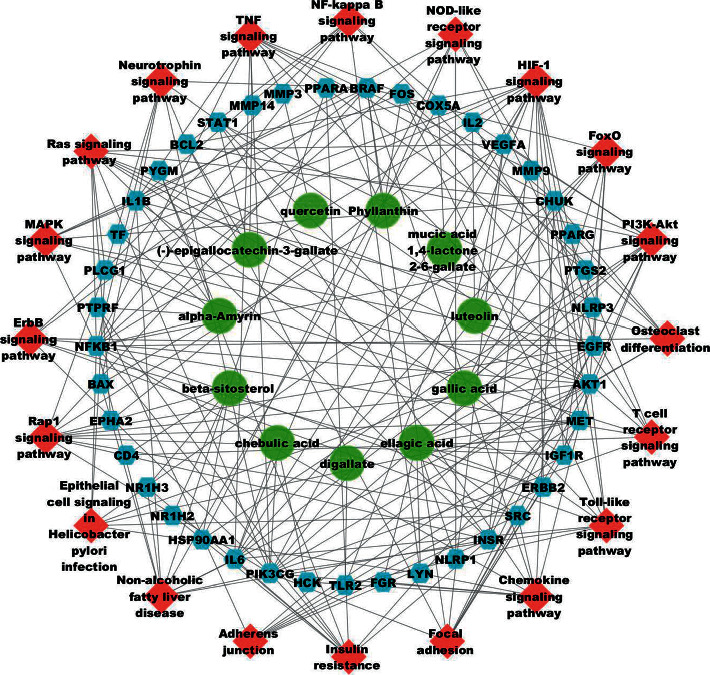
PEL component-target-pathway network diagram. The green circular nodes represent the active components of PEL, blue hexagons represent intersection targets, and red diamond nodes represent important paths. The edges represent the relationship among components, targets, and pathways.

PEL components, intersection targets, and important pathways were used to construct the CTP network of PEL with Cytoscape 3.7.0 (Figure 9). We consulted the literature to rule out the pathways that were not related to gout, such as cancer and hepatitis B, and selected the relatively important pathways [e.g., the HIF-1 (degree value: 15), PI3K-Akt (degree value: 12), TNF (degree value: 11), and NOD-like receptor signaling pathways (degree value: 7)] using the network analysis function in Cytoscape 3.7.0 (Table 3).

### 3.4. Mechanism Validation

#### 3.4.1. PEL Can Inhibit Inflammation in Gout Rats

**Figure 10:**
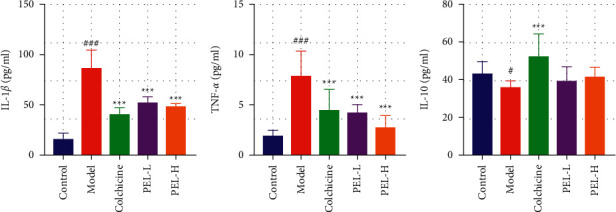
Changes in serum related factors of rats in each group after treatment (x̄ ± *s*, *n* = 9). The expression levels of proinflammatory cytokines IL-1*β* (a), TNF-*α* (b), and IL-10 (c). Compared with the blank group, ^#^*P* < 0.05, ^###^*P* < 0.0001; compared with the model group, ^*∗∗∗*^*P* < 0.001.

As shown in Figure 10, PEL significantly reduced the expression of the proinflammatory cytokines, IL-1*β* and TNF-*α*, and increased the expression of the anti-inflammatory factor, IL-10, in gout rats, although there was no significant difference in IL-10 expression between the Model and PEL groups.

#### 3.4.2. PEL Can Inhibit the Expression of MMP13 and NLRP3

**Figure 11:**
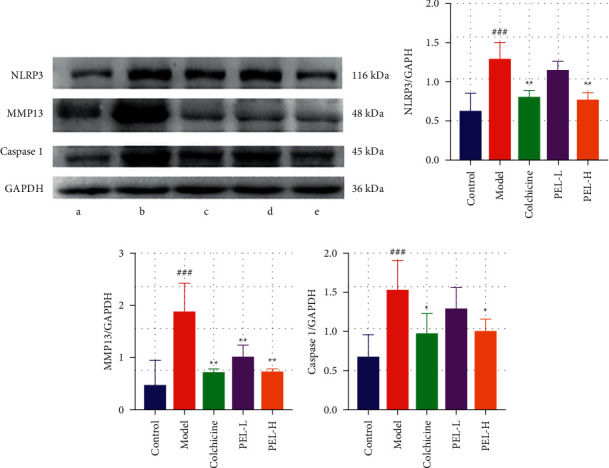
Changes in synovial protein and related factors in ankle joints of rats in each group after treatment. (a) The related factors in ankle joints of rats in each group, including a for Control group, b for Model group, c for Colchicine group, d for PEL-L group, and e for PEL-H group. The relative expression values of NLRP3 (b), MMP13 (c), and caspase-1 (d) in synovium of rat ankle joints were determined. Compared with the blank group, ^##^*P* < 0.01, ^###^*P* < 0.001; compared with the model group, ^*∗*^*P* < 0.05, ^*∗∗*^*P* < 0.01.

As shown in Figure 11, PEL-H significantly reduced the expression of NLRP3, MMP13, and caspase-1 in the ankle joint synovium. The expression of NLRP3, MMP13, and caspase-1 also decreased in the PEL-L group, but there was no significant difference in the expression of NLRP3 and caspase-1 between the PEL-L and Model groups.

## 4. Discussion

In the present study, the potential components and action targets of PEL in the treatment of AGA were analyzed by network pharmacology, and 11 components, 41 action targets, and 20 action pathways were found. Ellagic acid is one of the active components of PEL with suitable antioxidant and anti-inflammatory properties [35]. Gallic acid has also been shown to play a therapeutic role in inflammation and related diseases by targeting the MAPK and NF-*κ*B pathways [36]. The KEGG and CTP networks showed that PEL played a role in the treatment of AGA mainly through the HIF-1, PI3K-Akt, TNF, and NOD-like receptor signaling pathways.

Among these signaling pathways, HIF-1 has been confirmed to play an important role in hypoxic cells, while the latest research emphasizes the close relationship between hypoxia and inflammation. IL-1*β* is a key factor in acute gout inflammation [37]. Gupta et al. have shown that chitin can reduce the secretion of IL-1*β* by inhibiting the expression of HIF-1*α* and NLRP3 in macrophages, thus exerting therapeutic effects on AGA [38]. Related studies have also confirmed that HIF-1*α* can regulate the expression of NLRP3 in a venous thrombosis model [39]. This suggests that HIF-1*α* may have an effect on AGA by regulating the NLRP3 pathway. However, there are no studies to confirm that the HIF pathway has a definite effect on AGA; therefore, the complexity of the relationship between the HIF pathway and AGA needs to be studied further. The PI3K-Akt pathway plays an important role in the inflammatory response by activating NF-*κ*B, while NF-*κ*B is also one of the upstream signals for activating the NLRP3 inflammasome [40]. Related studies have shown that PI3K inhibitors can reduce neutrophil apoptosis and chondrocyte inflammation in osteoarthritic rats [41]. However, the pathogenesis of AGA through the PI3K-Akt pathway has not been completely elucidated to date.

Recent studies have shown that MSU deposition in the joints of patients with gout can trigger the formation of NLRP3 inflammasomes. NLRP3 inflammasomes activate IL-1*β* by activating caspase-1 and then activate the NOD receptor family signaling pathway to induce gout inflammation [42]. Given the essential role of the NLRP3/ASC/caspase-1 pathway in AGA development as well as the targets and pathways obtained using network pharmacological analysis, we finally selected the NLRP3 pathway to study the protective effect of PEL. In the present study, we established an experimental model of AGA by injecting rats with potassium oxycyanate and MSU. Unlike in humans, uricase is expressed in rats, and it results in the self-healing of acute inflammation in the AGA model within approximately three days [43]. Therefore, to obtain an adequate drug concentration in a short experimental time, each group of model rats was treated with corresponding drugs before and after injection with MSU.

At present, the pathological process of gout can be roughly divided into four stages: (1) hyperuricemia (HUA), but no MSU deposition or gout; (2) MSU deposition, but no gout symptoms; (3) MSU deposition accompanied by late AGA; (4) gout with various complications, such as gouty stone deposition, chronic gouty arthritis, and radiological erosion [44]. The clinical treatment of gout is mainly focused on reducing uric acid levels and inflammation in the third stage. The results of the current study showed that the alcohol extract of PEL could improve the appearance of ankle in the model group and reduce the gait score of gout rats and their degree of ankle swelling. These results show that the alcohol extract of PEL can reduce the pathological changes associated with gout arthritis in rats and exert a therapeutic effect on AGA.

Reducing uric acid levels plays a key role in the prevention and treatment of gout. The alcohol extract of PEL was confirmed to reduce uric acid levels in this experiment, and this result is consistent with that reported by Sato [15]. There are many reasons accounting for HUA. Excessive uric acid production or relatively insufficient uric acid excretion are the two main reasons for abnormal increase in uric acid levels in the body. As a key enzyme in purine metabolism pathway, XOD can convert hypoxanthine and xanthine into uric acid; this is also the most important pathway of uric acid production in vivo. We speculated that the alcohol extract of PEL could reduce uric acid levels by inhibiting the activity of XOD, which was successfully confirmed by measuring the activity of XOD in the serum of rats in each group. The American Rheumatic Society guidelines recommend that serum uric acid levels of all patients receiving uric acid–lowering therapy should remain below 360 *μ*mol/L or 6 mg/dL [45], because long-term levels below this critical level may dissolve MSU, inhibit inflammation, and eliminate gout [46]. However, the dosage of alcohol extract of PEL needed to achieve this uric acid–lowering effect in clinical settings still needs to be elucidated.

In AGA patients, the local inflammatory response induced by MSU deposited in the joint is a crucial cause of joint tissue injury. For example, colchicine, the positive drug used in this experiment, is one of the first-line drugs for the treatment of gout because of its outstanding anti-inflammatory effects [47, 48]. Our results show that the alcohol extract of PEL can reduce the severity of inflammatory reactions in gout rats, decrease the degree of neutrophil infiltration in the ankle joints of gout rats, and effectively improve various pathological conditions, such as synovial hyperplasia and hyperemia and joint structural disorder. Yang et al. demonstrated that the development of AGA is closely related to the NLRP3/ASC/caspase-1 pathway [49]. When the macrophages in the synovium of the ankle recognize the danger signal, which is the deposition of MSU in the joint cavity, through pattern recognition receptors (PRRs), the expression of NLRP3 inflammatory bodies is upregulated and posttranscriptional modification is carried out [50]. At this point, NLRP3 is activated, leading to the further recruitment of apoptosis-associated spot-like proteins (ASC), which then combines with caspase-aspartate protease (caspase-1) to form the NLRP3 inflammatory complex, cleaving the inactive caspase-1 into active cle-caspase-1 [51]. Cle-caspase-1 triggers the downstream inflammatory response, that is, the activation and release of interleukin-1 family proteins (e.g., IL-1) [52], followed by the production of several proinflammatory factors. These factors include TNF-*α*, IL-1*β*, chemokine monocyte chemoattractant protein-1, and macrophage inflammatory protein-1*α* produced by M1 macrophages to promote inflammation [53–55]. The results of the current study showed that the ethanol extract of PEL could significantly inhibit the expression of NLRP3 and caspase-1 and downregulate the expression of the proinflammatory cytokine IL-1*β*. This shows that PEL can be used for the treatment of AGA because it inhibits the activation and assembly of the NLRP3 inflammasome and regulates the production of downstream inflammatory factors. In the maintenance stage of inflammation, polarized M2 macrophages can secrete anti-inflammatory cytokines including TGF-*β* and IL-10, which may relieve gout inflammation [56]; however, the mechanism underlying spontaneous resolution has not been clearly elucidated. The alcohol extract of PEL could increase the level of IL-10 in gout rats, although there was no significant difference.

In addition, although the general process of NLRP3-mediated AGA is well understood, little is known about the upstream pathway that connects MSU crystals and NLRP3 activation. The two prerequisite steps for NLRP3 to mediate AGA are initiation and activation [57]. Activation occurs through the activation of nuclear factor NF-*κ*B by PRRs. The activation signal of NLRP3 is generated following the recognition of MSU by PRRs (mainly TLR4) [58, 59]. However, the exact pathway by which MSU activates NLRP3 is still unclear. It is suggested that the release of oxidative mitochondrial DNA, mitochondrial reactive oxygen species (ROS), and cardiolipin caused by K^+^-dependent or non-K^+^-dependent pathways is the main factor for the activation of the NLRP3 inflammasome [38]. Notably, the antioxidant activity of PEL is as significant as its anti-inflammatory activity. Nambiar et al. confirmed that PEL fruit extract has satisfactory antioxidant capacity through DPPH-free radical scavenging experiments [60], and Zhang et al. confirmed that PEL exerts a protective effect against H_2_O_2_-induced cellular injury [61]. We speculate that the ethanol extract of PEL may inhibit the activation of NLRP3 by scavenging ROS. It is believed that more experimental studies on the capacity of PEL for scavenging mitochondrial ROS in vivo in the future will not only reveal the internal relationships among different pharmacological activities of PEL but also enable an improved understanding of the pathogenesis of gout.

For patients with gout, osteoarthritis is a type of degenerative disease usually accompanied by inflammation, which eventually causes substantial damage to the articular cartilage. MMP13 is considered the key enzyme of cartilage degradation in osteoarthritis [62]. Compared with other proteins of the MMP family, the expression of MMP13 is markedly limited to connective tissue and has strong activity to degrade type II collagen in the cartilage [63]. It can degrade not only type II collagen in the cartilage but also proteoglycan, type IV collagen, and osteonectin [64], resulting in the destruction of the collagen network and the exposure of chondrocytes to inflammatory factors. Finally, under the action of the mechanical load and inflammatory factors, the apoptosis of chondrocytes occurs, which further aggravates the tissue injury of patients with gout. In view of the biological activity of MMP13, it has become an attractive target for the treatment of osteoarthritis, cancer, and cardiovascular diseases. However, safety and clinical efficacy are still two important problems to be solved for the clinical use of specific MMP13 inhibitors. The experiment described in the present study confirmed that the alcohol extract of PEL could effectively inhibit the expression of MMP13 in the synovium of the ankle joints of AGA rats. As a substance that can be used as both medicine and food, the safety of PEL is also relatively high [9]. Chaphalkar et al. found that PEL did not show any significant change in body weight and behavioral pattern in acute toxicity study [65]. Chaiyasut et al. also reported the safety of consuming Lactobacillus sp. mediated fermented PEL [66]. The resulting safer consumption of the PEE may be attributed to the presence of antioxidant and hepatoprotective activities [8]. This may provide novel insights into the treatment of osteoarthritis.

## 5. Conclusions

In this study, we confirmed that PEL extract can be used to treat gout as it reduces serum uric acid levels, inhibits inflammation, and protects ankle cartilage. PEL can reduce uric acid levels by inhibiting XOD activity, suppress the inflammatory response by inhibiting the NLRP3/caspase-1/IL-1 inflammatory axis, and protect ankle cartilage by inhibiting MMP13 expression. However, its mechanism of action and clinical dosage need to be further explored.

## Figures and Tables

**Table 1:** The UPLC-MS^n^ data and main compound names of the 5 peaks.

Peak no.	Scan mode	t R (min)	Molecular formula	[M ± H]	Identification	ppm	Identification
^a^1	Negative	0.81	C_6_H_10_O_8_	209.0307	MS1: 209.0307 [M − H]^−^, MS2: 191.0202 [M-H_2_O-H]^−^, 147.0302 [M-H_2_O-CO_2_-H]^−^	−0.48	Mucic acid
^a^2	Negative	0.88	C_6_H_8_O_7_	191.0202	MS1: 191.0202 [M − H]^−^, 383.0477 [2M − H]^−^ MS2: 147.0302 [M-CO_2_-H]^−^	0.52	Mucic acid lactone
^a^3	Negative	1.61	C_7_H_6_O_5_	169.0149	MS1: 169.0149 [M − H]^−^, MS2: 125.0251 [M-CO_2_-H]^−^	0.59	Gallic acid
^b^4	Positive	20.68	C_24_H_39_O_4_	391.2838	MS1: 391.2838 [M + H]^+^	−0.51	Ethyl hexyl phthalate
^c^5	Negative	2.26	C_6_H_12_O_6_	179.0564	MS1: 179.0564 [M − H]^−^, MS2: 101.0248 [C_4_H_5_O_3_]^−^, 89.0242 [C_3_H_5_O_3_]^−^, 71.01382 [C3H_2_O_2_]^−^	−2.78	Glucose

a: Gallotannins, b: Phthalates, c: Glucose.

**Table 2:** Basic information on the active compounds of PEL.

MOLID	Compound	CAS number	MW	OB (%)	DL
MOL006812	Phyllanthin	10351-88-9	418.58	33.31	0.42
MOL000006	Luteolin	491-70-3	286.25	36.16	0.25
MOL000358	Beta-sitosterol	83-46-5	414.79	36.91	0.75
MOL006824	*α*-Amyrin	638-95-9	426.8	39.51	0.76
MOL000422	Kaempferol	520-18-3	286.25	41.88	0.24
MOL001002	Ellagic acid	476-66-4	302.2	43.06	0.43
MOL005983	Leukoefdin	491-52-1	322.29	43.45	0.31
MOL000098	Quercetin	117-39-5	302.25	46.43	0.28
MOL006793	Mucic acid 1, 4-lactone 2-0-gallate	—	358.28	49.56	0.31
MOL006821	(-)-Epigallocatechin-3-gallate	989-51-5	458.4	55.09	0.77
MOL000569	Digallate	536-08-3	322.24	61.85	0.26
MOL006826	Chebulic acid	23725-05-5	356.26	72	0.32
MOL000513	Gallic acid^*∗*^	149-91-7	170.13	31.69	0.04

**Table 3:** Degree value analysis of pathways in CTP network.

Pathways	Betweenness centrality	Closeness centrality	Degree
HIF-1 signaling pathway	0.10670956	0.44099379	15
PI3K-akt signaling pathway	0.06644801	0.4251497	12
TNF signaling pathway	0.13508328	0.42011834	11
Ras signaling pathway	0.03548477	0.41520468	11
Rap1 signaling pathway	0.03281526	0.41040462	11
Nonalcoholic fatty liver disease	0.09957878	0.41040462	10
Insulin resistance	0.08416535	0.40571429	10
Focal adhesion	0.02776726	0.40112994	10
Chemokine signaling pathway	0.06689398	0.41040462	10
Toll-like receptor signaling pathway	0.04355976	0.40571429	9
T-cell receptor signaling pathway	0.04668759	0.40112994	8
Osteoclast differentiation	0.0411098	0.39664804	8
FoxO signaling pathway	0.01449155	0.39226519	8
NOD-like receptor signaling pathway	0.03845429	0.37566138	7
NF-kappaB signaling pathway	0.02278709	0.38378378	7
Neurotrophin signaling pathway	0.01906849	0.38797814	7
MAPK signaling pathway	0.01459004	0.38797814	7
ErbB signaling pathway	0.01213661	0.38378378	7
Epithelial cell signaling in helicobacter pylori infection	0.01438965	0.38378378	7
Adherens junction	0.01115371	0.33023256	7

## Data Availability

The data that support the findings of this study are openly available.

## Abbreviations

ASC: Apoptosis-associated spot-like proteins
AGA: Acute gouty arthritis
BP: Biological processes
CC: Cellular components
Colchicine: Colchicine group
Control: Control group
CTP: Component-target-pathway
DL: Drug-like
HE: Hematoxylin and eosin
MCODE: Molecular complex detection
MF: Molecular functions
Model: Model group
MSU: Monosodium urate
OB: Oral bioavailability
PEL: *Phyllanthus emblica* L.
PEL-L: Low-dose PEL-treated group
PEL-H: High-dose PEL-treated group
PPI: Protein-protein interaction
PRR: Pattern recognition receptors
ROS: Reactive oxygen species
SD: Sprague Dawley
XOD: Xanthine oxidase
UA: Uric acid
SPF: Specific-pathogen-free
HUA: Hyperuricemia.
