# Supplementary figures and images for: Exploring the Mechanism through which Phyllanthus emblica L. Extract Exerts Protective Effects against Acute Gouty Arthritis: A Network Pharmacology Study and Experimental Validation
Source: Evid Based Complement Alternat Med. 2022 Apr 11;2022:9748338. doi: 10.1155/2022/9748338 (PMC9017503; doi:10.1155/2022/9748338)

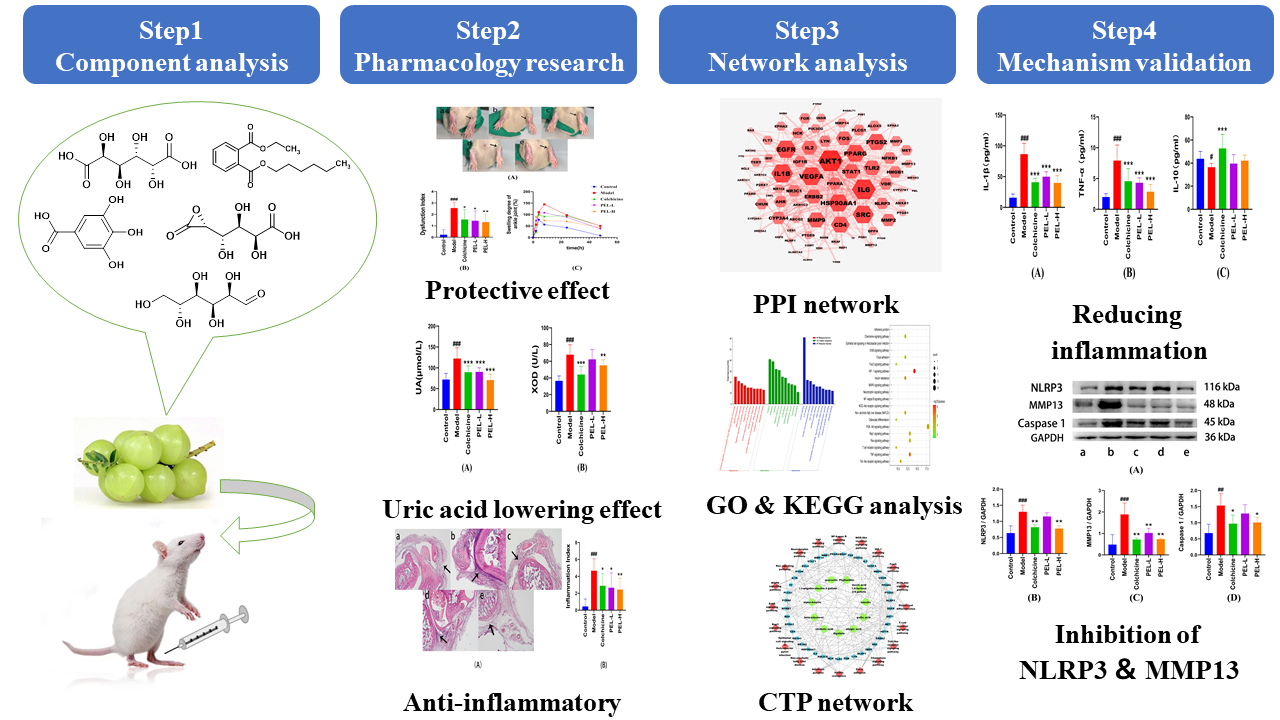

Supplement: Supplementary Materials — The graphical abstract is provided to clarify the main strategies of the research. [file 9748338.f1.docx]
